# Simulation framework for connected vehicles: a scoping review

**DOI:** 10.12688/f1000research.73398.1

**Published:** 2021-12-09

**Authors:** Siti Fatimah Abdul Razak, Sumendra Yogarayan, Afizan Azman, Mohd Fikri Azli Abdullah, Anang Hudaya Muhamad Amin, Mazzar Salleh

**Affiliations:** 1 Faculty of Information Science and Technology, Multimedia University, Ayer Keroh, Melaka, 75450, Malaysia; 2 Kolej Universiti Islam Melaka, Kuala Sg Baru, Melaka, Malaysia; 3 Faculty of Computer, Information Science and Applied Media, Higher Colleges of Technology, Dubai, United Arab Emirates; 4 Rolls-Royce, Derby, England, UK

**Keywords:** V2V, network simulator, mobility generator, simulations, connected vehicles, microscopic models

## Abstract

**Background: **V2V (Vehicle-to-Vehicle) is a booming research field with a diverse set of services and applications. Most researchers rely on vehicular simulation tools to model traffic and road conditions and evaluate the performance of network protocols. We conducted a scoping review to consider simulators that have been reported in the literature based on successful implementation of V2V systems, tutorials, documentation, examples, and/or discussion groups.

**Methods: **Simulators that have limited information were not included. The selected simulators are described individually and compared based on their requirements and features, i.e., origin, traffic model, scalability, and traffic features. This scoping review was reported according to the Preferred Reporting Items for Systematic Reviews and Meta-Analyses extension for Scoping Reviews (PRISMA-ScR). The review considered only research published in English (in journals and conference papers) completed after 2015. Further, three reviewers initiated the data extraction phase to retrieve information from the published papers.

**Results: **Most simulators can simulate system behaviour by modelling the events according to pre-defined scenarios. However, the main challenge faced is integrating the three components to simulate a road environment in either microscopic, macroscopic or mesoscopic models. These components include mobility generators, VANET simulators and network simulators. These simulators require the integration and synchronisation of the transportation domain and the communication domain. Simulation modelling can be run using a different types of simulators that are cost-effective and scalable for evaluating the performance of V2V systems in urban environments. In addition, we also considered the ability of the vehicular simulation tools to support wireless sensors.

**Conclusions: **The outcome of this study may reduce the time required for other researchers to work on other applications involving V2V systems and as a reference for the study and development of new traffic simulators.

## Introduction

In recent decades, a significant increase in vehicle use has increased traffic congestion and fatalities ^1^. According to the World Health Organization, 1.25 million people are killed and severely injured involving vehicle accidents ^2^. Hence, connected vehicle technology responds to this constraint, aiming to leverage inter-vehicle communication to produce safe, user-friendly, and fuel-efficient vehicle assistive technologies ^3, 4^. One of the main aspects of connected vehicle research is to optimise traffic flow through the exchange of information ^5^. This communication can be sorted in terms of vehicle (V2V), infrastructure (V2I), a pedestrian (V2P), and network (V2N) ^6, 7^. The exchange of information, collectively known as V2X communications, could assist drivers in preventing accidents by providing warnings of danger invisible to drivers and other sensors (e.g. collision avoidance, lane departure warning and speed limit alert) ^8, 9^.

Nevertheless, the adoption of connected vehicle technology poses a range of challenges, particularly in urban environments. It is challenging to analyse the effectiveness of the application of connected vehicles under traffic conditions ^10– 12^. As such, simulations using traffic and network simulators as well as mobility generators are viable alternatives to modelling and determining the effectiveness of such deployments in the real world ^13, 14^, as it provides an affordable and scalable method for analysing model compliance in various contexts and parameters.

Traffic simulations are categorised by level of detail into three separate categories ^15^. First, the most precise information on each vehicle in the system is microscopic simulations ^16^. Second, mesoscopic simulations exploit aggregate velocity-density functions to represent their behaviour and view traffic as a continuous stream of vehicles ^17^. Finally, macroscopic simulation is the large-scale traffic model, which focuses on combined traffic status ^18^. Microscopic simulations provide the highest degree of detail for modelling, although they are the slowest to execute ^19, 20^.

In addition, mobility generators are a possible option for modelling vehicle elements such as traffic, temporal and spatial mobility, and generating mobility traces ^21, 22^. These traces are then uploaded to a network simulator, which mimics vehicle-to-vehicle communication. Furthermore, these traces can be generated by observing real-world vehicles on the road and then used in network simulations ^23, 24^. The effect of network parameter modifications on traffic mobility is a strategic objective simulation ^25^. It is also restricted to the use of the trace controlled by the mobility model. Another option is to use a simulator that directly integrates the mobility framework.

For Vehicular Adhoc Networks (VANET), it is necessary to rely on network protocols to assess their performance ^22, 23^, given that actual experiments are not possible. Over the last decade, efforts have been made to produce a full transport simulator for VANET solutions, including a wireless network simulator for modelling and evaluation ^24, 25^. A wide range of simulators can be used for VANET simulation modelling, both commercial and open source. Older simulators provide a network simulator to communicate with stationary mobility models. Many researchers have examined various mobility models with simulation tools for several contexts. Such simulator tools are not yet well explored since many researchers base their simulations depending on their use case settings. Thus, this motivates the identification of different simulators do not yet exist. Therefore, this study conducted a systematic scoping review to identify the applicability and availability of existing mobility generators, network simulators, and combination simulators.

## Methods

**Figure 1.**
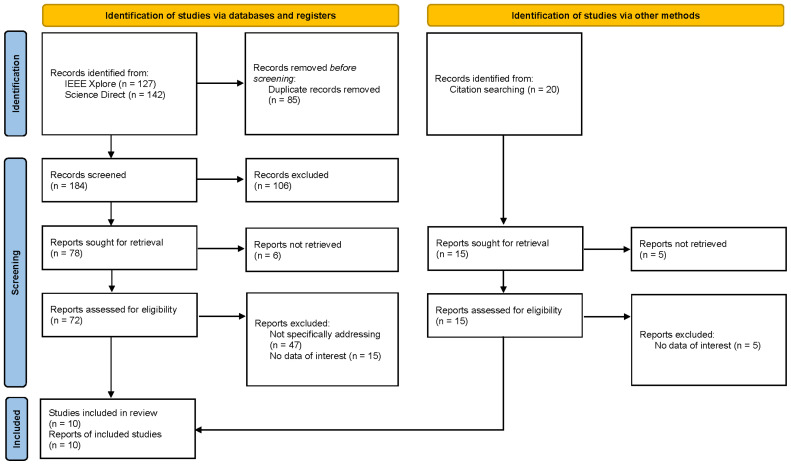
PRISMA Flow Diagram.

A popular approach to synthesize research evidence which have no definitive procedure established is known as a scoping review. To conduct the review process, we adopted the PRISMA Extension for a Scoping Review ^26^. The process involves determining the subjective and objective outcomes, identifying and selecting relevant studies, organising and summarising the findings, and reporting the outcomes (see Figure 1). Review questions were developed as follows:

1. What are available mobility generators which are currently active in vehicle simulations?
2. What is the criteria of network simulators commonly used in vehicle simulation active development?
3. Which mobility networks and network simulators have been integrated to study vehicle communication protocols?

### Search, sources and inclusion criteria

Relevant studies were identified from IEEE and ScienceDirect, only including journal and conference papers with a published status. All searches were initiated in November 2019, and articles published in English starting from 2015 were considered for evaluation as our analysis of the literature shows that it is a relatively new but rapidly growing field of academic endeavour.

Controlled phrases and free-text word phrases related to vehicular communications that investigated V2V safety applications, vehicle network performance, driver behaviour, vehicle simulation tools, and VANET were considered for inclusion (see Box 1).

Mendeley was used to import the search results ( www.mendeley.com) and remove duplicate articles.

**Box 1. Search string**

"VANET"

#1 AND Simulation

#1 AND #2 AND Simulators

#1 AND #2 AND #3 AND Routing

#1 AND #2 AND #3 AND #4 AND Mobility

#1 AND #2 AND #3 AND #4 AND #5 AND Urban

### Screening

Once duplicate articles had been removed, further screening was performed at the title or abstract level based on the inclusion criteria. In addition, related technical reports were also included from Google Scholar. For confirmation, filtering (e.g. possible synonyms or other related terms) was also done at the full-text level afterwards. Citations of the articles included were searched for other relevant articles.

To facilitate the screening task, we imported the bibliographic citation file in RIS format from Mendeley into Rayyan ( https://www.rayyan.ai/). Three reviewers (S.Y., A.A., and M.F.A.A.) were involved in the screening process to minimise bias and ensure the consistency of the selected articles. The first screening process ended in April 2020 and was later updated in January 2021.

### Data extraction and analysis

All reviewers reviewed the same article and reports during the data extraction phase before collating their findings in an MS Excel spreadsheet. Data related to objective outcomes were collected from each included article wherever available, including year, type of traffic model, architecture and simulation language, type of network simulator, type of mobility generator, implementation or experimentation scenario and type of license. The spreadsheets were compared to ensure consistent data extraction by all reviewers. The contribution of the studies was further analysed based on descriptions provided in the publications paper to consider in our scoping review. In March 2021, the data extraction and analysis were finalised by consensus among the reviewers.

## Results

A total of 269 publications were found initially. After removing duplicates, a total of 184 titles and abstracts were screened, from which 72 publications were subjected to full-text review after excluding those not of interest to this study. In total, ten studies and reports fulfilled the criteria for inclusion and were included in the analysis (see Figure 1).

We found that open-source mobility and network simulators were popular among researchers. Microscopic models were preferable for research related to vehicular communications since the simulations provide the most precise information of each vehicle or mobile node and the highest degree of detail for modelling compared to macroscopic and mesoscopic models. Common network simulators were NS-2, Ns-3 and OMNeT++. However, not all mobility simulators supported active development, which is important in current active research domains such as vehicular communications. The summary of mobility generators and network simulators found are in Table 1 and Table 2, respectively. A list of all ten studies can be found in Table 3.

**Table 1.** Mobility generators.

Reference(s)	Name of mobility generator	Active development	Release	License	Map	Traffic model	Network simulator
27– 29	SUMO	Y	2021	Open Source	Real and User Defined	Microscopic Mesoscopic	NS-2, NS-3, OMNeT++
30– 32	MATSim	Y	2021	Open Source	Real and User Defined	Microscopic	N/A
33	DTALite	Y	2021	Open Source	Real	Mesoscopic	N/A
34, 35	SMARTS	Y	2020	Open Source	Real and User Defined	Microscopic	N/A
21, 36, 37	PARAMICS	Y	2020	Commercial	Real and User Defined	Microscopic	NS-2, OMNeT++
31, 37	MovSim	Y	2018	Open Source	Built-In	Microscopic	N/A
21, 38, 39	VISSIM	Y	2016	Commercial	Real and User Defined	Microscopic Mesoscopic	NS-2, QualNet
40	VNEtIntSim	N	2015	Open Source	Real and User Defined	Microscopic	Integration OPNET
37	Traffisim	N	2014	Open Source	Real and User Defined	Microscopic	N/A
41	CityMob	N	2009	Open Source	Built-In	Microscopic Macroscopic	NS-2
41	FreeSim	N	2008	Open Source	Real	Microscopic Macroscopic	N/A
41	STRAW	N	2007	Open Source	Built-In	Microscopic	NS-2, SWANS
41	Vanet- MobiSim	N	2007	Open Source	Real and User Defined	Microscopic	NS-2, QualNEt, OMNeT++, GloMoSim

Y = Supported, N = Not Supported

**Table 2.** Network simulators.

Reference(s)	Name of network simulators	Active development	Release	License	802.11p Support	Architecture Language	Simulation Language
42, 43	OPNET	Y	2021	Commercial	Y	C++	C++ OTCL
32, 42, 44	NS-3	Y	2021	Open Source	Y	C++ Python	C++ Python
42	OMNeT++	Y	2020	Open Source	Y	C++	C++
32, 42	QualNet	Y	2019	Commercial	Y	C++	C++
42, 45	NS-2	N	2011	Open Source	Y	C++	C++ OTCL
32, 42	JiST/SWANS	N	2005	Open Source	N	JAVA	JAVA
40, 45	GloMoSim	N	2000	Open Source	N	C	C

Y = Supported, N = Not Supported

**Table 3.** Previous studies.

Reference	Contribution	Scenario	Protocol Used	Mobility Simulator	Network Simulator	Simulator and Framework
46	This paper provides a comparison of three routing protocols in the VANET scenario. The result focuses on determining the effectiveness of routing protocols for several performance measures of which the vehicle is an essential aspect of the evaluation.	Urban	DSDV AODV DSR	SUMO MOVE	NS-2	N/A
47	The paper provides a simulation in the VANET scenario at a vast scale. The result is focused on the performance of four routing protocols under different checks in terms of delay, packet delivery, overhead, and transmission power.	Urban	OLSR DSDV AODV DSR	SUMO	NS-3	N/A
48	This paper uncovers an automatic routing protocol for the VANET scenario. The idea is to disseminate the information provided by several roadside units. There are three routing protocols evaluated using several performance metrics in terms of delay, number of hops, total service time, and number of fragments.	Urban	ARP GSR A-STAR	SUMO	OMNeT++	N/A
49	The paper focuses on two routing protocols within the VANET scenario. The idea is to ensure an optimal path from source to destination under a few performance measures in terms of throughput and packet delivery ratio.	Generic	DYMO OLSR	N/A	QualNet	N/A
50	This paper investigates DSRC 5.9 GHz for the V2V scenario in restricted areas. The findings were reviewed using three routing protocols using different performance parameters in terms of delay and number of forwarding nodes.	Generic	EMDV >MHVB EDB	N/A	NetSim	N/A
29	The paper provides an analysis of four routing protocols within the VANET scenario. The outcome was assessed based on a different mobility model and speed and performance parameters such as goodput, throughput, packet receive performance and receive rate.	Urban	OLSR AODV DSDV DSR	SUMO	NS-3	N/A
27	This paper provides a comparison of three routing protocols for the VANET scenario. The results show the performance in the transmission of critical information within the framework of several performance assessments in terms of goodput and packet delivery ratio.	Generic	OLSR AODV DSDV	SUMO	NS-3	N/A
51	The paper presents a fuzzy logic method to improve the routing protocol in the VANET scenario. The study demonstrated the simulation by considering the number of vehicles, the extent of the transmission, and vehicle speed movement.	Urban Generic	AODV	SUMO	OMNeT++	N/A
52	This paper uncovers a road recovery mechanism in the VANET scenario. The study improved the pathway to better message delivery by considering mobility measures such as relative speeds and relative distance.	Generic	CLARR CCBR	N/A	NS-2	N/A
53	The paper examined two routing protocols for better message dissemination in V2V and V2I scenarios. The findings demonstrated optimised routing under several performance assessments like throughput, packet loss, packet delivery report, and delay.	Urban	AODV DSR	SUMO	N/A	NetSim

N/A – Not Applied

## Discussion

Since this area of study is considered as a relatively new but rapidly growing field, this scoping review process only considers relevant papers published from 2015 onwards, which shows that extensive research has been conducted to create security standards for communication technologies, particularly the vehicular network. Although various simulators can be enhanced with library extensions, none of the simulators is related to security and privacy. Ultimately, researchers and professionals cannot compare their security measures to a given circumstance. For instance, ensuring the privacy of a vehicular user in a fast-moving network and disseminating messages in a secure vehicular environment. However, there is no simple practice of extending existing simulators to the desired security standard, which implies that future development research will need to be done.

In addition, the quality of a simulation depends largely on the precision of the models. The range of precision has increased dramatically recently, where several modules contain signal attenuation components, multiple antenna models, and environmental interferences. However, one continuous barrier to producing accurate simulations is the evolution of rapid prototyping and its increasing use in-vehicle networks. For example, vehicle nodes would depend on three-dimensional scenarios to communicate with other nodes. It would be crucial for current and future simulators to extend the current simulators to these new conditions.

Integration with real-time system modelling based on non-real-time events creates additional challenges. Due to resource limitations, current simulators do not correspond with the physical properties of the hardware prototype while simulating a comprehensive network with multiple vehicles. Several alternatives have been put forward to reduce the complexity that could speed the simulation. However, this approach usually does not include indirect outcomes, which could seriously impact the behaviour of real-world network components. It is, therefore, necessary to examine the interconnection between simulators and hardware devices with the security standards concerned.

## Conclusions

Studies have led to the discovery of comprehensive and realistic simulation tools due to the increasing popularity and interest for the future transportation system. This work has examined the current availability of simulators. Although several simulators have many features, it is worth exploring further the improvement of the simulators for specific scenarios.

## Data availability

### Underlying data

All data underlying the results are available as part of the article and no additional source data are required.

### Reporting guidelines

Zenodo: PRISMA-ScR checklist for ‘Simulation framework for connected vehicles: a systematic review’, https://doi.org/10.5281/zenodo.5637802 ^54^

Data are available under the terms of the Creative Commons Attribution 4.0 International license (CC-BY 4.0).
