# Supplementary material for: Simulation framework for connected vehicles: a scoping review
Source: F1000Res. 2023 Feb 16;10:1265. Originally published 2021 Dec 9. [Version 2] doi: 10.12688/f1000research.73398.2 (PMC9958301; doi:10.12688/f1000research.73398.2)
Supplement: Peer review history

# Peer review: Reviewer response for version 2

**DOI:** 10.5256/f1000research.143645.r163702

**Published:** 2023-02-24

**Authors:** Lionel Nkenyereye

**Affiliations:** 1 Department of Computer and Information Security, Sejong University, Seoul, South Korea

I have carefully reviewed the revised version of the article. Therefore, the authors addressed all my comments. The paper is well shaped to the acceptance decision.

Are the rationale for, and objectives of, the Systematic Review clearly stated?

Yes

Is the statistical analysis and its interpretation appropriate?

Yes

Are sufficient details of the methods and analysis provided to allow replication by others?

Yes

Are the conclusions drawn adequately supported by the results presented in the review?

Partly

Reviewer Expertise:

Vehicular technology, edge computing, and software-defined networks

I confirm that I have read this submission and believe that I have an appropriate level of expertise to confirm that it is of an acceptable scientific standard.

# Peer review: Reviewer response for version 2

**DOI:** 10.5256/f1000research.143645.r163703

**Published:** 2023-02-23

**Authors:** Mahmoud Zaki Iskandarani

**Affiliations:** 1 Al-Ahliyya Amman University, Amman, Jordan

There seems to be major improvements in the submitted work.

Are the rationale for, and objectives of, the Systematic Review clearly stated?

Yes

Is the statistical analysis and its interpretation appropriate?

Partly

Are sufficient details of the methods and analysis provided to allow replication by others?

Yes

Are the conclusions drawn adequately supported by the results presented in the review?

Partly

Reviewer Expertise:

Intelligent Transportation Systems, Artificial Intelligence, Mobile and wireless Communication, Sensors and systems.

I confirm that I have read this submission and believe that I have an appropriate level of expertise to confirm that it is of an acceptable scientific standard.

# Peer review: Reviewer response for version 1

**DOI:** 10.5256/f1000research.77046.r158671

**Published:** 2023-01-04

**Authors:** Mahmoud Zaki Iskandarani

**Affiliations:** 1 Al-Ahliyya Amman University, Amman, Jordan

The article attempts to produce a guidance into the most appropriate simulators for V2V, and in general V2X communications. This effort is a good effort and in the right direction in terms of what is witnessed of V2X developments under cooperative driving. However, the authors can benefit from the following comments:

1. Assigning the most appropriate simulator for a V2V or V2X application with in depth correlative analysis linked to different scenarios and protocols.
2. Comparative analysis of the used simulators per specific published article or group of articles sharing common objective, and the extent of benefit and development achieved using such simulator.
3. More detailed discussion and more comprehensive conclusion and recommendation will greatly improve the article.

Are the rationale for, and objectives of, the Systematic Review clearly stated?

Yes

Is the statistical analysis and its interpretation appropriate?

Partly

Are sufficient details of the methods and analysis provided to allow replication by others?

Yes

Are the conclusions drawn adequately supported by the results presented in the review?

Partly

Reviewer Expertise:

Intelligent Transportation Systems, Artificial Intelligence, Mobile and wireless Communication, Sensors and systems.

I confirm that I have read this submission and believe that I have an appropriate level of expertise to confirm that it is of an acceptable scientific standard, however I have significant reservations, as outlined above.

# Response

**Published:** 2023-01-26

**Authors:** Siti Fatimah Abdul Razak

**Affiliations:** Multimedia University, Malaysia

The applicability of V2V or V2I (V2X) has been added in Table 2 (page 7).

 The comparative analysis has been highlighted in Table 3 (page 7).

 The conclusion has been revised as suggested (page 9).

# Peer review: Reviewer response for version 1

**DOI:** 10.5256/f1000research.77046.r143137

**Published:** 2022-07-19

**Authors:** Lionel Nkenyereye

**Affiliations:** 1 Department of Computer and Information Security, Sejong University, Seoul, South Korea

The study surveys the existing simulation framework for connected vehicles. The works proposed a method based on PRISMA Extension for a Scoping Review. The previous study table is short but relevant contributions are presented. These contributions summarize the routing concept, dissemination of the information, and performance measures in terms of throughput and packet delivery ratio.

 The following comments could enhance the quality of this work:

1. In the conclusion, I request the authors to specify the type of mobility generator and mobility network that are efficient for real-time system modelling.
2. At the Screening section, authors focus much on VANET. How about other vehicular network that support V2V? For instance software-defined based VANET or vehicular Edge/Fog technology or Vehicular Cloud network in their Search string. Is the current work includes different type of vehicular technology in general? Therefore, rewrite in the introduction that this study focuses particularly on VANET.

Are the rationale for, and objectives of, the Systematic Review clearly stated?

Yes

Is the statistical analysis and its interpretation appropriate?

Yes

Are sufficient details of the methods and analysis provided to allow replication by others?

Yes

Are the conclusions drawn adequately supported by the results presented in the review?

Partly

Reviewer Expertise:

Vehicular technology, edge computing, and software-defined networks

I confirm that I have read this submission and believe that I have an appropriate level of expertise to confirm that it is of an acceptable scientific standard, however I have significant reservations, as outlined above.

# Response

**Published:** 2023-01-26

**Authors:** Siti Fatimah Abdul Razak

**Affiliations:** Multimedia University, Malaysia

The paper is mainly focusing on VANET deployment. The introduction has been revised as suggested (page 2 and 3). 

 The conclusion has been revised to specify the type of mobility generator and mobility network that are efficient for real-time system modelling (page 9).
